# Molecular Tools and Schistosomiasis Transmission Elimination

**DOI:** 10.4269/ajtmh.20-0111

**Published:** 2020-08-10

**Authors:** Joseph Hamburger

**Affiliations:** Department of Microbiology and Molecular Genetics, The Kuvin Center for the Study of Infectious and Tropical Diseases, The Hebrew University of Jerusalem, Hadassah Medical School, Jerusalem, Israel

## Abstract

Large-scale control efforts in sub-Saharan Africa may leave long-term lingering transmission. Large-scale screening of snail infection prevalence by loop-mediated isothermal amplification will enable accurate determination of man-to-snail transmission, as well as the effects of biota in snail habitat on host capacity and thus on snail-to-man transmission. Next-generation sequencing will enable identification of gut content of snails and thus their feeding preferences in hot spots and in non–hot spots, as well as for identification of attractive vegetation types for attracting snails to molluscicides.

## INTRODUCTION

Schistosomiasis, afflicting more than 240 million people,^1^ is undergoing control across much of south Saharan Africa (SSA).^2,3^ Complete interruption of transmission of schistosomiasis was also targeted in selected regions by 2025.^4,5^ Snail control added to public chemotherapy alone^6^ was applied quite successfully even before the onset of mass administration of praziquantel (PZQ),^7^ and its importance is increasingly recognized.^8^

In certain SSA countries, schistosomiasis transmission may linger for a considerable time after repeated mass control activities. Its duration can be expected to depend on human activities and on control input, which may require examination of additional concepts and technologies.

## MEDICAL ASPECTS

These came first in control efforts, given existing mass infection and morbidity, which required mass diagnosis, initially by detecting ova in excreta and subsequently by detecting circulating antigens (circulating anodic antigen [CAA] and circulating cathodic antigen [CCA]) in blood and urine.^9–11^ This was later used for large-scale screening of the *Schistosoma mansoni* infection rate by using reagent strips.^12^ Reduction of infection and morbidity was achieved by repeated mass drug administration (MDA), but it did not stop transmission. This was achieved only in a limited part of the wide distribution area but not in SSA, where infection is the most prevalent.^2–4,6,13^ Moreover, reduction of prevalence can return in some areas to high levels when stopping repeated MDA.^14,15^

## SCHISTOSOMIASIS TRANSMISSION ELIMINATION

Vaccination is considered a preferred approach for this purpose, and investigational efforts continue on this line, but an effective and applicable human vaccine is not yet available.^16^ Alternative means are based on human behavior, clean water supplies, and facilities for removing sewage.

The most sought-after control means were those directed toward reduction of snail populations.^17^ Niclosamide, available commercially as Bayluscide, is used for large-scale mollusciciding,^18^ and in certain areas, when combined with the use of PZQ treatment led to an enhanced control effect.^6,19–21^ Introduction of certain species of crayfish feeding on snails^22–24^ was also successful in eliminating snails, but a large-scale application requires further investigation in various types of water bodies. Removal of vegetation on which snails feed was also attempted successfully^25,26^ but cannot be considered for large-scale control.

Mass screening of snail infection rate is required as long as transmission continues, and the loop-mediated isothermal amplification (LAMP) was suggested for this purpose^27^ and later actually tested for large-scale screening.^28^ Loop-mediated isothermal amplification will identify all infected snails, as did PCR, whether shedding cercariae or not.^27,28^ Identification of snail-to-man transmission, and vice versa, traditionally relied on the identification of snails shedding cercariae. Identification of cercarial shedding is still required for identification of transmission spots from snails to man, especially hot spots (see the following texts). If LAMP is established for large-scale and repeated detection of infected snails, the relation between diagnosis in snails and in humans will require reexamination for determining the desired timing of each. Also, the strategy for diagnosis in humans with different levels of water contact^13,29^ requires reevaluation toward replacing mass diagnosis and MDA by a more focused choice of target groups.

## LOOKING CLOSER AT SNAILS

Factors which affect transmission and its elimination require a closer look at snails and on biota coexisting with them in the habitat. Satellite-based remote sensing contributed to a broad outlook on factors affecting transmission, including water flow, water temperature, and coverage by vegetation.^30^ Drone-based remote sensing was used to identify vegetation types, and floating and nonemergent vegetation types shown to cause formation of snail clusters were detected. This provided a habitat proxy for high transmission and suggested the effect of vegetation on transmission intensity.^8^ In the future, development of drone-carried sensors suitable for detecting snails’ life signals may enable a direct mass identification of snail density in transmission sites. This will enable targeting snails by molluscicides, as reported for identifying mosquito larvae, hosts of *Plasmodium*.^31^ Until this is realized, other approaches for focal snail control must be sought (as suggested in the following).

When environmental factors affecting transmission by snails take the lead in control activities, snails show characteristics of a pest and should be treated as such. The example of *Trichobilharzia* spp. present in many water resorts can be more easily perceived as a pest causing cercarial dermatitis in humans.^32^ But snails transmitting schistosomes are no less pests than mosquitoes transmitting malaria parasites, in terms of intensive human exposure under natural living conditions. Pest control is usually a repeated struggle for controlling the density of pests, preferably with the participation of the affected population (see the following texts).

## MASS DETECTION OF INFECTED SNAILS BY LAMP^27,28^

This is very simple to apply if the complex amplification mixtures used for it (including DNA polymerase, amplification primers, nucleotides, and buffer components) are provided to field laboratories ahead of time and stored until used.^27^ Determination of prevalence by testing pools of snails will further facilitate these tests.^28^ Loop-mediated isothermal amplification is particularly important for identifying lingering or resurgent transmission, with a low prevalence of infected snails. Differentiation between snails infected by human schistosomes and those infected by animal schistosomes is also possible by molecular tools.^33,34^ PCR, the molecular tool preceding LAMP,^35,36^ was usually rejected for field studies, as it depended on high and expensive technology and on molecular biology know-how.

## SNAIL-TO-MAN TRANSMISSION CAPACITY

This appears to be affected by snails’ habitat and can now be identified by using molecular tools. Previously, it was reported that certain species of vegetation and other biota in snails’ habitat can affect transmission to man. It was, thus, shown that water lilies^37^ and rice^38^ affect snail abundance and support increased cercarial numbers in the corresponding sites. On the other hand, attachment of rotifers to snails inhibits development of schistosomes within them and reduces the viability of cercariae.^39,40^ In the case of inhibited development of schistosomes within snails, the availability of molecular monitoring of infected snails at prepatency^35^ will enable the detection of factors affecting host capacity in the laboratory and in nature. In practice, cercarial shedding is the indicator of the actual snail-to-man transmission. However, without molecular screening, for detecting host capacity of snails, the lack of cercarial shedding can be mistaken for cessation or reduction of man-to-snail transmission.

A year-long screening of infected snails was carried out in which snails were collected at 2-week intervals and examined by both cercarial shedding and by PCR.^41^ Infected snails were identified by PCR in every snail batch collected from all sites (a total of 19 sites in coastal Kenya) for the whole year, but in several sites, detection by cercarial shedding was not shown at all during this period, or was very rare. This study brings to our attention a condition of “cold spots,” as opposed to the well-known hot spots of transmission (see the following texts). The prevalence of such “cold spots” in endemic areas and their effect on transmission intensity need to be examined at multiple sites, and at shorter time intervals, before and after application of MDA. This is required to evaluate how common this phenomenon is and for seeking out environmental factors that may affect its formation.

Although the matter of “cold spots” needs to be further examined and established, the reality of hot spots is known for years and recognized as a very important factor responsible for persistent transmission after MDA.^42^ Measurement of cercarial shedding can reveal the presence of hot spots where infected snails shedding cercariae are present in especially large numbers and human infection rates are higher than those in nearby “non–hot spots” after MDA.^8,42^ When looking at transmission intensity in a wide sense, the terms hot spots, non–hot spots, and “cold spots” are expressions of snail-to-man transmission intensity. “Cold spots” are actually non–hot spots with especially low cercarial shedding, and the clarification of this situation requires further molecular testing of snail capacity in hot spots and in a range of non–hot spots, where different rates of cercarial shedders are found. Various factors relating to nearby human populations were mentioned regarding the formation of hot spots,^8,42^ and information on the relations between snails and their habitat (types of vegetation) in promoting hot spots was recently addressed.^8^ Also, the detection of snail clusters by counting snails in the environment^8^ can suggest attraction of snails to preferred vegetation types, which can be regarded as relevant for developing the concept of the bait principle for focused mollusciciding. Further search of environmental predictors of persistent hot spots was recently presented,^43^ based on further environmental factors and related factors. Although the vicinity of transmission sites to agricultural plots was mentioned in this study, the identity of the vegetation types in these plots and the possibility that any of them reached the concerned transmission sites and struck roots were not elaborated on. The use of the relatively new molecular tool, the new-generation sequencing (NGS), can be useful for identifying the gut contents of snails, as was performed in sand flies, hosts of *Leishmania*,^44^ and thus provide answers to the feeding preferences of snails from each of the spots mentioned earlier.

## A FOCUSED APPLICATION OF MOLLUSCICIDES

This is required for increasing mollusciciding efficiency by directing it to sites with high snail abundance. Feeding preference of snails, as determined by NGS, opens up the gate for examining the bait principle for attracting snails to a mollusciciding environment. Testing of this principle in the laboratory clearly demonstrated that the combination between L-glutamine and a slow release polymeric formulation (calcium alginate beads) of niclosamide had a strong attraction and mollusciciding effect.^45^ In addition to L-glutamine, several other amino acids were also identified as attractive for snails (cited in Refs. 45 and 46), and certain other compounds such as starch and glycogen were also found to be attractive for snails.^47^ These experiments indicated that the combination between an attractant (bait) and niclosamide does not deter snails from being attracted and then killed. In other experiments, it was further shown that *Biomphalaria glabrata* snails are attracted or trapped by minced lettuce,^48^ thus demonstrating the potential of vegetation items as snail baits. The combination of attractive vegetation and molluscicides can be expected to be less costly and easier to produce for application on a large scale. Such a formula is potentially suitable as “pest control” used with the participation of exposed populations.

Agricultural plants with leaves have leftovers which may be available for examining their attraction for snails. The information on vegetation preference by snails awaits laboratory studies comparing snails growing on Romanian lettuce, the common feeding vegetation in the laboratory, with a variety of agricultural products that may be considered as potential baits. If indeed attractive, these vegetation types when distributed in suitable spots are expected to cause clustering of snails around them. The use of NGS^44,49^ can provide identification of snails’ feeding preferences. The potential usefulness of a bait–niclosamide combination requires that molluscicides can be attached to attractive vegetation species, as is being widely applied in agriculture for attaching fertilizers.^50^ Bait-based control can be considered to be sound as a combination of attractive L-arginine and niclosamide does not deter snails from being attracted to the molluscicide.^45^ Various possible industrial products of vegetation–molluscicide combination can be considered, according to optimal distribution and efficacy strategies.
